# Dynamics of reduced genetic diversity in increasingly fragmented populations of Florida scrub jays,*Aphelocoma coerulescens*

**DOI:** 10.1111/eva.13421

**Published:** 2022-06-01

**Authors:** Tram N. Nguyen, Nancy Chen, Elissa J. Cosgrove, Reed Bowman, John W. Fitzpatrick, Andrew G. Clark

**Affiliations:** ^1^ Department of Ecology and Evolutionary Biology Cornell University Ithaca New York USA; ^2^ Cornell Lab of Ornithology Ithaca New York USA; ^3^ Department of Biology University of Rochester Rochester New York USA; ^4^ Department of Molecular Biology and Genetics Cornell University Ithaca New York USA; ^5^ Avian Ecology Lab Archbold Biological Station Florida USA

**Keywords:** habitat loss, heterozygosity, immigration, inbreeding, population decline, runs of homozygosity

## Abstract

Understanding the genomic consequences of population decline is important for predicting species' vulnerability to intensifying global change. Empirical information about genomic changes in populations in the early stages of decline, especially for those still experiencing immigration, remains scarce. We used 7834 autosomal SNPs and demographic data for 288 Florida scrub jays (*Aphelocoma coerulescens*; FSJ) sampled in 2000 and 2008 to compare levels of genetic diversity, inbreeding, relatedness, and lengths of runs of homozygosity (ROH) between two subpopulations within dispersal distance of one another but have experienced contrasting demographic trajectories. At Archbold Biological Station (ABS), the FSJ population has been stable because of consistent habitat protection and management, while at nearby Placid Lakes Estates (PLE), the population declined precipitously due to suburban development. By the onset of our sampling in 2000, birds in PLE were already less heterozygous, more inbred, and on average more related than birds in ABS. No significant changes occurred in heterozygosity or inbreeding across the 8‐year sampling interval, but average relatedness among individuals decreased in PLE, thus by 2008 average relatedness did not differ between sites. PLE harbored a similar proportion of short ROH but a greater proportion of long ROH than ABS, suggesting one continuous population of shared demographic history in the past, which is now experiencing more recent inbreeding. These results broadly uphold the predictions of simple population genetic models based on inferred effective population sizes and rates of immigration. Our study highlights how, in just a few generations, formerly continuous populations can diverge in heterozygosity and levels of inbreeding with severe local population decline despite ongoing gene flow.

## 1 INTRODUCTION

Habitat loss and fragmentation are the greatest threats to biodiversity today (Brooks et al., 2002; Groom et al., 2012; Hanski, 2005, 2011), in large part because these landscape changes reduce connectivity among populations, causing local population declines and concomitant reduction in genetic diversity leading to extirpation (Frankham, 2005; Hedrick, 2001; Ouborg et al., 2006). The loss of heterozygosity and elevated frequency of deleterious alleles can lead to inbreeding depression (Gao & Gao, 2016; Kawamura, 2005; Liberg et al., 2005; Lynch et al., 1995), lowered resistance to diseases (Anderson & May, 1986; Pearman & Garner, 2005; Spielman et al., 2004; Tarpy, 2003; Whiteman et al., 2006), and increased susceptibility to environmental fluctuations (Charlesworth & Charlesworth, 1987; Pearman & Garner, 2005), all of which increase extinction risk. Furthermore, standing genetic variation within populations is required for species to adapt to environmental change by providing the raw materials for evolution (Barrett & Schluter, 2008). Thus, understanding the genomic consequences of population decline in the wild, especially the pace of genomic changes during early stages of decline, is critical for conservation management and for predicting the vulnerability of species to habitat loss and intensifying global change.

A prevailing conservation genetics paradigm holds that shrinking, isolated populations are more susceptible to genetic drift and stochastic processes that reduce genetic variation and increase homozygosity (Frankham, 2005; Hedrick, 2001; Lynch et al., 1995; Soulé & Simberloff, 1986). Additionally, as populations decline, the probability of inbreeding increases, causing individuals to become more genetically similar to one another and often decreasing individual fitness (Charlesworth, 2009; Charlesworth & Charlesworth, 1987). Despite these theoretical predictions, empirical evidence about the scale and pace of genetic changes remains scarce, particularly in populations with persistent immigration, such as in structured metapopulations, and especially in the early stages of decline as connected subpopulations begin to differentiate (Kohn et al., 2006; Ouborg et al., 2006).

The Florida scrub jay (*Aphelocoma coerulescens*; FSJ) is an endemic, nonmigratory bird that exists in metapopulations scattered across the Florida peninsula (Stith et al., 1996) and currently faces severe population decline (Boughton & Bowman, 2011; Coulon et al., 2012). FSJs are cooperative breeders that live in family groups consisting of breeding pairs, many of which retain a few offspring as helpers. The FSJ depends on early successional, fire‐maintained xeric oak scrub habitat that was once widespread across the peninsula but has declined and become fragmented over the last century due to anthropogenic habitat conversions to citrus plantations, housing developments, and fire suppression. By the early 1990s, the state‐wide FSJ population had declined by >90%, leaving only about 10,000 individuals scattered across hundreds of local demes state‐wide (Boughton & Bowman, 2011; Stith et al., 1996).

**FIGURE 1:**
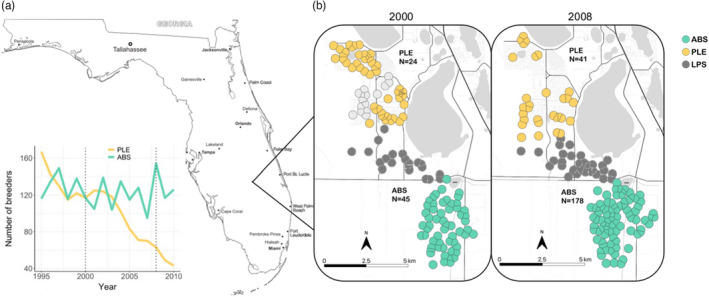
(a) Change in the number of breeders recorded in ABS (green) and PLE (yellow) from 1995 to 2010. Samples for this study collected in 2000 and 2008—indicated with dotted lines—incorporate early and precipitous late stages of the population decline in PLE. (b) Thiessen polygons around sampling localities in 2000 and 2008. Green and yellow polygons represent sampled localities in ABS and PLE, respectively. Gray polygons denote unsampled but extant territories within the metapopulation, including LPS denoted with dark gray

Here, we capitalize on this unique opportunity to study two neighboring subpopulations that were recently continuous but are now experiencing contrasting demographic conditions. An intensively studied subpopulation of FSJs in wild habitat at Archbold Biological Station (ABS) in south‐central Florida has remained stable over 50 years because of consistent land protection and management, while a nearby subpopulation within the same metapopulation at Placid Lakes Estates (PLE) has declined precipitously over the last decade because of habitat loss to residential housing development and fire suppression (Figure 1). We sought to quantify genomic changes associated with local habitat loss and sudden population decline within a subpopulation still connected by limited gene flow to nearby stable populations. Our objective was to understand how quickly genomic impacts accumulate as population size and immigration rate decrease. We used single‐nucleotide polymorphism (SNP) and pedigree data from each population at two time points, 2000 and 2008—approximately two generations spanning the early‐to‐late stages of population decline—to quantify changes in levels of genetic diversity, inbreeding, and degree of relatedness within and between our two focal subpopulations over time.

## 2 MATERIALS AND METHODS

### 2.1 Study subpopulations

ABS harbors one of the largest and best protected subpopulations of FSJs within its 2200 ha of suitable, ecologically managed oak scrub. Due primarily to prescribed burning for habitat management, the FSJ subpopulation at ABS has remained relatively stable in size and trajectory, ranging between 95 and 154 breeders from 1995 to 2010. Since 1969, a study population within ABS has been color banded for individual recognition and familial relationships were documented in a population pedigree allowing for accurate ascertainment of immigrants coming into the population (Woolfenden & Fitzpatrick, 1984). Every year, all nests within each FSJ family group are found and monitored, every territory is mapped, and every nestling is measured, banded, and a blood sample is taken.

Less than 10 km northwest of ABS, another subpopulation of FSJs exists within a residential subdivision, PLE (Figure 1b). The ABS and PLE subpopulations were continuous and panmictic in relatively intact habitat as recently as the 1980s, but habitat loss and fragmentation increased within PLE during that decade. These subpopulations remain contiguous through Lake Placid Scrub (LPS), an unsampled, wildland subpopulation of FSJs that was restored during our sampling period. Banding data confirm that limited gene flow existed between ABS and PLE during our sampling interval. Previous research using microsatellite markers placed these subpopulations within the same genetic unit and metapopulation (Coulon et al., 2008), and our current SNP data also confirm low divergence between the two populations (*F*_ST_ = 0.008). While median natal dispersal in FSJs is typically less than 1 km (Aguillon et al., 2017), birds occasionally move farther (Suh et al., 2020). During our sampling interval, dispersal of birds from the native ABS to the suburban PLE was very rare (*N* = 3), while dispersal in the reverse direction (suburban to native) was an order of magnitude more common (Bowman & Woolfenden, 2001).

Despite their connectivity and close proximity, ABS and PLE are experiencing drastically different population trajectories. Beginning in the 1980s, oak scrub habitat within PLE existed within a mosaic of private ownerships that were being cleared and occupied by humans as suburbanization intensified. Annual monitoring in PLE began in 1991 and continued through 2011. Although some population decline likely occurred in the 1980s with the initial onset of development, the PLE study tract contained sufficient habitat to support a population comparable to, and continuous with, ABS until the 1990s. Field monitoring estimated 115 breeders in PLE during 1998, equivalent to ABS at that time (Figure 1a). Supplemental feeding associated with the suburbs in PLE during the 1980s likely maintained this high breeder count. However, after 1990, as the residential development of PLE accelerated, suitable habitat steadily disappeared, immigration rates into PLE plummeted, and the subpopulation declined precipitously.

### 2.2 Sample availability and genotyping

We genotyped existing blood samples from individuals in ABS and PLE in the years 2000 and 2008. We sampled only breeders to avoid biasing genetic estimates by including individuals known or suspected to be closely related (e.g., from within the same nuclear family). In 2000, we have samples from 45 breeders at ABS and 24 breeders at PLE. In 2008, we obtained samples from 178 breeders in ABS and 41 breeders in PLE.

Blood was obtained from the brachial vein in each bird's wing via needle prick and DNA was extracted for subsequent genotyping at Geneseek (Neogen, Inc.) using a custom‐designed Illumina iSelect BeadChip covering 15,416 genome‐wide SNPs (Chen et al., 2014, 2016). SNP quality control (Gentrain score > 0.7, SNP and individual call rate > 95%) and pedigree validation were performed using GenomeStudio (Illumina), PLINK (Purcell et al., 2007), and PedCheck (O'Connell & Weeks, 1998) resulting in a dataset of 11,737 autosomal SNPs in Hardy–Weinberg equilibrium used in our runs of homozygosity analyses. Z‐linked and SNPs with high linkage disequilibrium were then pruned in PLINK (options ‐‐indep‐pairwise 50 5 0.2), resulting in a final dataset of 7834 autosomal SNPs for 288 breeding individuals.

### 2.3 Heterozygosity, inbreeding, and relatedness

Because cryptic population structure within ABS and PLE may influence downstream analyses exploring the genetic attributes of our study populations, we first searched for genetic clusters within the populations using principal component analysis (PCA; Figure S1) performed with the R package SNPRelate (Zheng et al., 2012). We found no evidence of hidden structure within our subpopulations and proceeded with subsequent genetic analyses. Because genetic drift is expected to impact allele frequencies in small, declining populations more than in large, stable ones, we plotted the observed changes in allele frequencies in ABS and PLE from 2000 to 2008 (Δ*p*) and compared the variances (Figure S2). We also ran simulations to explore the magnitude of changes in allele frequencies between ABS and PLE prior to the year 2000 with varying population sizes and increasing isolation between the two subpopulations (see Figure S3 and Supplemental Methods).

We contrasted levels of genetic diversity, inbreeding, and relatedness of breeders between subpopulations and across the sampling periods using PLINK (options ‐‐het, ‐‐ibc, ‐‐homozyg, and ‐‐genome), and tested for significance using the Wilcoxon rank‐sum test in R (R Core Team, 2015; Wickham, 2009). We estimated mean site‐based heterozygosity per individual in PLINK, defined as the number of heterozygous loci out of the total loci genotyped in that individual. Because we genotyped our samples using Illumina BeadChips, it is important to note that our study represents heterozygosity only at this subset of known SNP locations and is not the average per‐nucleotide heterozygosity across the entire genome.

To estimate levels of inbreeding for each individual, we used PLINK's designation of *F* ^III^ (Purcell et al., 2007; Yang et al., 2011), as it has been shown to have a strong correlation with pedigree‐based inbreeding coefficients (Chen et al., 2016; Huisman et al., 2016). We also used the proportion of the genome in runs of homozygosity (*F*_ROH_) because *F*_ROH_ is correlated with homozygous mutation load and thus is informative for studies of inbreeding depression (Kardos, Åkesson, et al., 2018a). We also estimated the number and lengths of runs of homozygosity (ROH) across the genome. The lengths of ROH, contiguous tracts of homozygosity arising from consanguineous mating which are broken up over time by recombination (Franklin, 1977), are particularly useful for anchoring the timing of inbreeding events. While shorter ROH could arise from background relatedness or linkage disequilibrium, longer ROH (on the order of several megabases)—as predicted for the early stages of population decline at PLE—reveals more recent inbreeding events (Pemberton et al., 2012; Thompson, 2013). Below, we briefly describe our ROH investigation.

We identified ROH segments and calculated *F*_ROH_ using our entire dataset of 11,737 autosomal SNPs that passed quality filters (see Supplemental Methods). We used a sliding window approach in PLINK (option ‐‐homozyg) and did not do linkage disequilibrium pruning for this analysis as it often reduces detection of ROH and thus biases estimates of *F*_ROH_ (Meyermans et al., 2020). To account for the low density of our BeadChip markers along the genome (average distance between adjacent SNPs ~85.5 kb; Figure S4), we adjusted the required minimum ROH length and average SNP density per ROH (see Supplemental Methods). Given our SNP sparsity and these parameters, we only had the ability to detect large (≥1 Mb) ROH. To account for different sample sizes, we performed 1000 iterations in which we randomly subsampled 24 and 41 individuals from ABS (to match the sampling in PLE) for the years 2000 and 2008, respectively. We then compared the mean number of ROH segments discovered in the ABS subsample to our observed PLE estimates (Figure S5). We also accounted for sampling disparities in ROH lengths by reporting these estimates as relative proportions within each subpopulation.

Finally, we also detected ROH using a model‐based method implemented through the program BCFtools/RoH (Narasimhan et al., 2016)—as opposed to the genotype counting method implemented by PLINK—and compared our results across the two methods. We found consistent significant trends for every analysis across the two programs, suggesting that our defined PLINK thresholds for ROH detection gave equivalent results to model‐based models. Thus, all ROH results henceforth are reported using our PLINK results. For details on our ROH analyses and comparison of results between the methods, see Figures S4, S5, and Supplemental Methods.

To assess degree of relatedness, we calculated pairwise identity by descent (IBD; Purcell et al., 2007) and compared mean IBD within and between subpopulations in 2000 and 2008. We also quantified the proportion of specific relationship classes (parent–offspring, full siblings, half siblings, avuncular, grandparent–grandchild, and first cousins or greater) in each subpopulation (see Supplemental Methods). We defined pairwise IBD bins for each relationship class based on the range of observed pairwise IBD values for known relationships in the pedigree, with the minimum considered pairwise IBD of 0.09 corresponding to the lower end of observed pairwise IBD for known first cousins. See Supplemental Methods for more details on our relatedness analyses.

## 3 RESULTS

### 3.1 Mean site‐based heterozygosity and inbreeding levels

**FIGURE 2:**
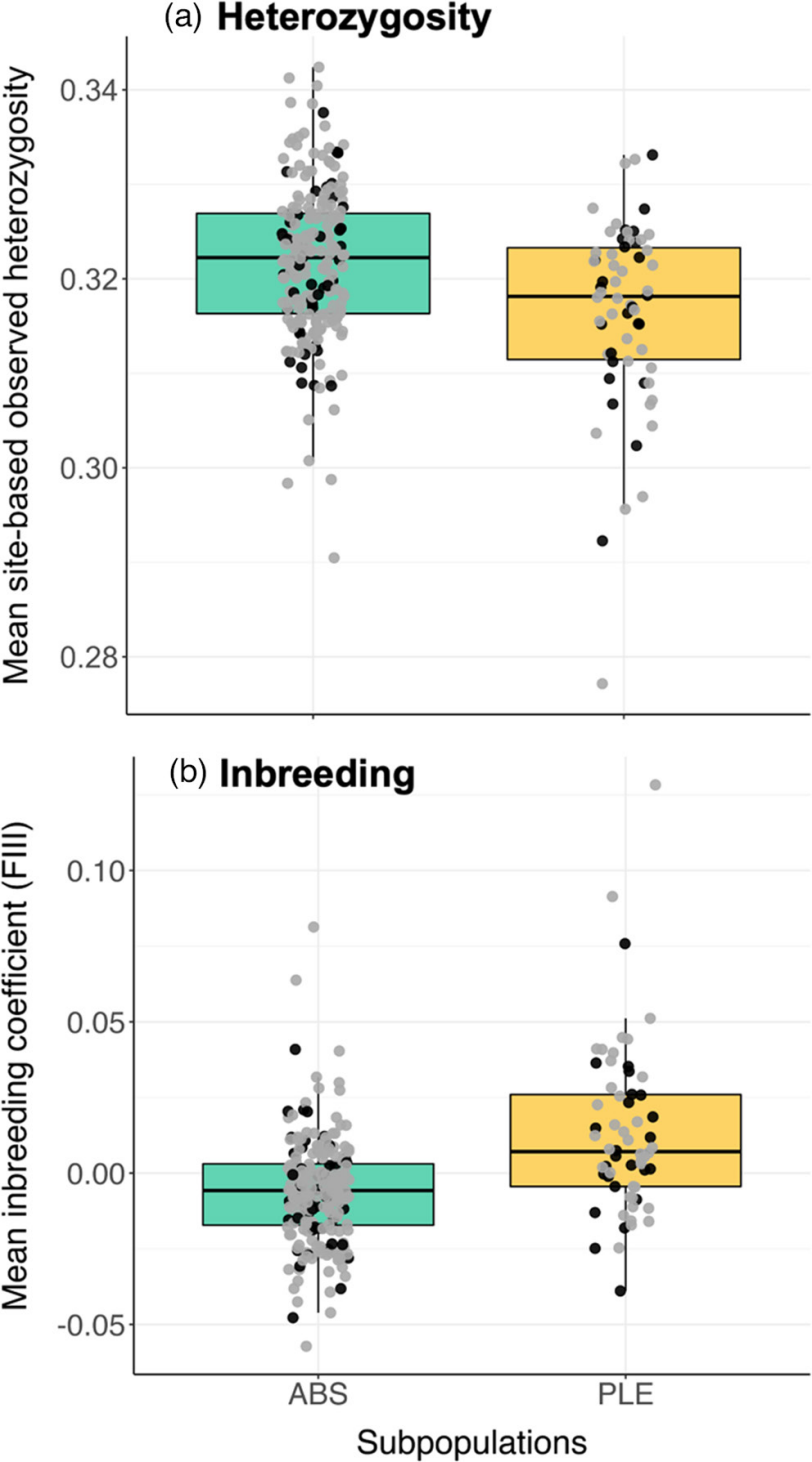
Boxplots for (a) average site‐based heterozygosity (*p* = 1.1 × 10^−4^) and (b) mean inbreeding coefficient F^III^ (*p* = 1.9 × 10^−8^) for ABS and PLE pooled across both years. The bottom, bolded middle, topmost lines of the boxes represent the first quartile, mean, and third quartile, respectively. Individuals represented as black and gray points for 2000 and 2008, respectively

We compared mean site‐based heterozygosity between subpopulations and across our sampling interval. As predicted, birds in ABS were more heterozygous than birds in the collapsing PLE subpopulation in both 2000 and 2008 (Table 1; Wilcoxon rank‐sum test: *W* = 729.5, *p* = 0.009, and *W* = 4689, *p* = 0.002, respectively). We detected no significant temporal change in mean site‐based heterozygosity when comparing within either subpopulation between 2000 and 2008. Therefore, we pooled our samples and found that ABS was still significantly more heterozygous than PLE (Figure 2a; Wilcoxon rank‐sum test: *W* = 8761, *p* = 1.00 × 10^−4^). Consistent with our heterozygosity results, the larger apparent effective size of the ABS population compared to PLE is also reflected in the lower variance in changes in SNP allele frequencies (from 2000 to 2008) in ABS compared to PLE (Figure S2).

**TABLE 1:** Mean values for genetic attributes for ABS and PLE in 2000 and 2008

Variable	ABS 2000 (*N* = 45)	PLE 2000 (*N* = 24)	ABS 2008 (*N* = 178)	PLE 2008 (*N* = 41)	ABSvPLE 2000 (*N* = 69)	ABSvPLE 2008 (*N* = 219)
Site‐based heterozygosity	0.323 (0.001)	0.317 (0.002)	0.322 (0.006)	0.316 (0.001)		
Inbreeding (F^III^)	−0.006 (0.003)	0.009 (0.005)	−0.006 (0.001)	0.014 (0.005)		
Inbreeding (FROH)	0.005 (1.0 × 10^−4^)	0.008 (0.002)	0.006 (6.2 × 10^−4^)	0.012 (0.003)		
Relatedness (pairwise IBD)	0.026 (0.002)	0.041 (0.004)	0.028 (4.8 × 10^−4^)	0.034 (0.002)	0.024 (0.001)	0.024 (3.4 × 10^−4^)

*Note*: Standard errors presented in parentheses.

To compare levels of inbreeding between our subpopulations and across the sampling interval, we estimated inbreeding coefficients *F* ^III^ and *F*_ROH_. We found that *F* ^III^ was significantly lower in ABS than PLE in both 2000 and 2008 (Table 1; Wilcoxon rank‐sum test: *W* = 757, *p* = 0.003 and *W* = 5253, *p* = 5.83 × 10^−6^, respectively), again consistent with predictions based on rapid population decline in PLE. Because we detected no significant changes in *F* ^III^ across 2000 and 2008 for either subpopulation, we again compared the *F* ^III^ statistic between ABS and PLE as pooled samples (Figure 2b; Wilcoxon rank‐sum test: *W* = 9765, *P* = 1.91 × 10^−8^). *F*_ROH_ was significantly lower in ABS than in PLE for both years (Table 1; Wilcoxon rank‐sum test: *W* = 285.5, *P* = 0.03 in year 2000 and *W* = 2668.5, *P* = 0.042 in 2008), and the two inbreeding statistics were significantly correlated (Figure S6; Pearson's correlation test: *r* = 0.57, *P* = 2.2 × 10^−16^).

As another assessment of the degree of inbreeding, we compared the number of ROH across subpopulations and sampling years. Overall, we found ROH on 28 of the 32 chromosomes in which we had SNP markers available (Figure S7). In 2000, we recorded 82 and 63 ROH segments in ABS and PLE, respectively. In 2008, we saw an increase to 328 and 104 segments in ABS and PLE. We accounted for the greater number of individuals in ABS than PLE (especially in 2008) by subsampling ABS to match our PLE sample sizes and instead compared the distribution of ROH counts generated for the subsample of ABS to the observed estimates in PLE. The mean number of ROH segments found in our subsampled ABS distribution was 43.84 ± 0.19 for 2000 and 79.59 ± 0.32 in 2008, which is consistently lower than the number of ROH detected in PLE for either year (Figure S5; Wilcoxon rank‐sum test: *W* = 0, *P* = 0.041 in year 2000 and *W* = 6, *P* = 0.043 in year 2008).

**FIGURE 3:**
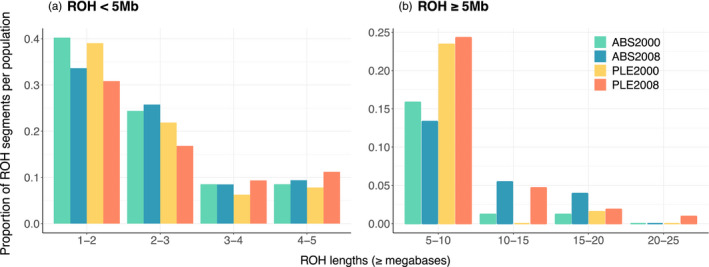
Proportion of ROH lengths in ABS and PLE using 11,737 SNPs. (a) Because recombination events break up ROH over time, runs shorter than 5 mb indicate linkage disequilibrium or inbreeding events in the distant past, while (b) ROH longer than 5 mb reveal more recent inbreeding

Because we expected more recent inbreeding events and, thus, longer ROH associated with habitat loss and fragmentation in PLE, we investigated the distribution of lengths of ROH <5 Mb and ≥5 Mb for both subpopulations and sampling periods. We selected this 5 Mb division based on similar empirical studies of inbreeding (Bosse et al., 2012; Humble et al., 2020). Short ROH such as those arising from background relatedness or linkage disequilibrium typically measure tens of kilobases in length (Pemberton et al., 2012). Therefore, we suspect that our cut‐off at 5 Mb, while arbitrary, truly reflects long ROH occurring from recent shared parentage. For this analysis, we did not subsample ABS to correct for sampling disparities but instead compared the ROH lengths as proportions within each subpopulation (Figure 3). The majority of segments were <5 Mb, with similar proportions across ABS and PLE. However, for ROH ≥5 Mb, the proportions in PLE were larger than in ABS across both years, consistent with our predictions for a continuously declining subpopulation in PLE. Specifically, these proportions were 0.18 in ABS and 0.24 in PLE in the year 2000 (Pearson's Chi‐square test: *X* ^2^ *=* 1.07, *df* = 1, *p* = 0.30), and 0.22 compared to 0.32 in 2008 (Pearson's Chi‐square test: *X* ^2^ *=* 4.04, *df* = 1, *p* = 0.04). Notably, the longest segment (nearly 25 Mb), and hence the most recent inbreeding event, was found in PLE in 2008. Finally, as with site‐based heterozygosity and *F* ^III^, we detected no significant changes in ROH lengths from 2000 to 2008 within either subpopulation.

### 3.2 Relatedness and identity by descent

**FIGURE 4:**
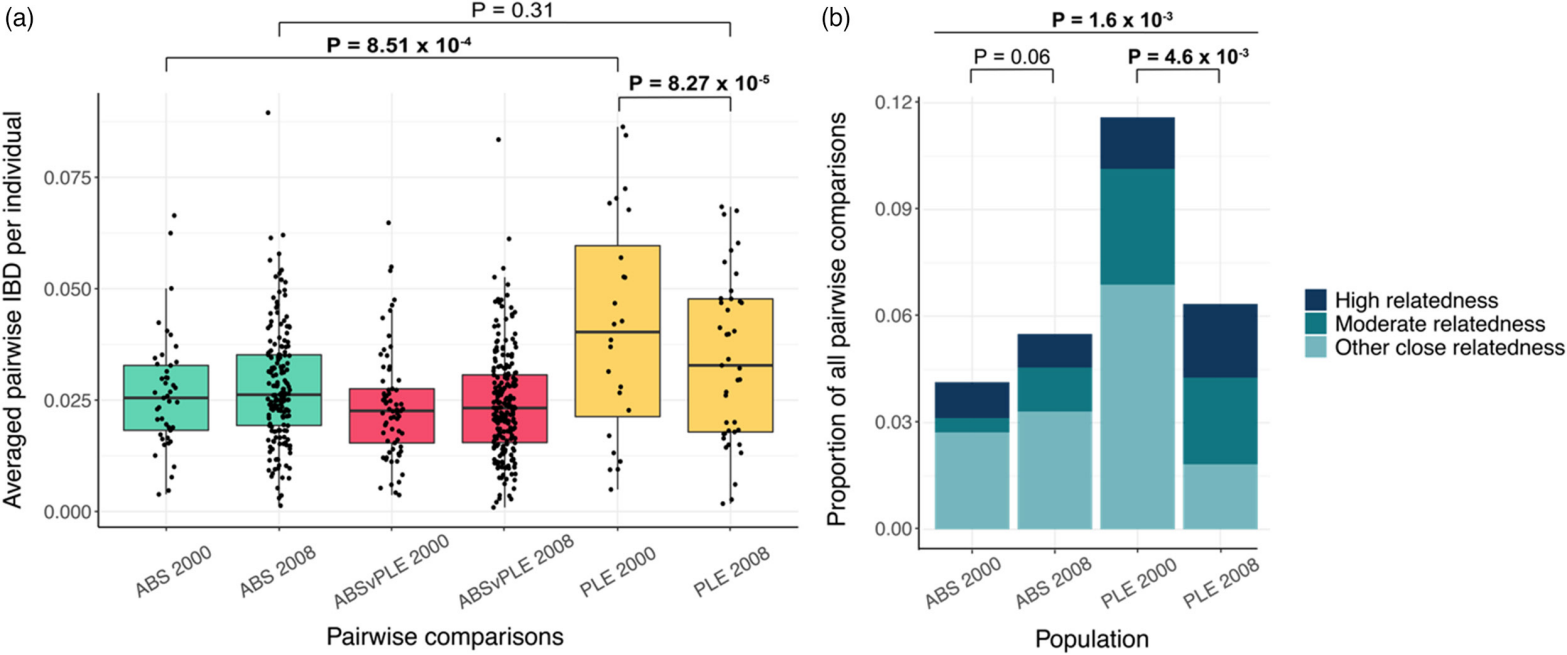
(a) Average pairwise identity by descent (IBD) of individuals in ABS and PLE in 2000 and 2008. The bottom, bolded middle, topmost lines of the boxes represent the first quartile, mean, and third quartile, respectively. Consistent with a large, stable population, mean IBD was lower within ABS than PLE for both years, although this was only statistically significant in 2000. As expected, mean pairwise IBD relatedness between subpopulations (ABSvPLE) was lower than within subpopulation comparisons for either year. (b) Proportions of the closest pairwise relationships (those with shared IBD ≥0.09 indicating first cousins or greater) within each subpopulation for 2000 and 2008. Thresholds for pairwise IBD bins for each relationship class were based on pairwise IBD values of known relationships in the pedigree. The high relatedness class (IBD ≥0.40) includes parent–offspring and full sibling pairs; the moderate relatedness class (IBD ≥0.18–0.40) includes half sibling, avuncular, and grandparent–grandchild pairs; and the other close relatedness class includes first cousin pairs (IBD ≥0.09–0.18). As expected, ABS had a smaller overall proportion of close relationships than PLE in both years. PLE also had a significantly larger proportion of the high and moderate relatedness classes than ABS for both years. Across the sampling interval, the overall proportions did not significantly change in ABS (from 0.041 to 0.055) but decreased in PLE (from 0.115 to 0.063), driven primarily by first cousin pairs. This decrease in close relationship pairs within PLE reflects the decline in pairwise IBD observed from 2000 to 2008

As population size declines and consanguineous matings increase, individuals are expected to become more related to one another over time. We assessed the degree of relatedness within ABS and PLE and across our sampling periods using a pairwise IBD approach. Consistent with predictions for a large, stable population, individuals in ABS had lower average pairwise IBD values than individuals in PLE in either year, although this comparison was only statistically significant for 2000 (Figure 4a; Table 1; Wilcoxon rank‐sum test: *W* = 152,700, *p* = 8.51 × 10^−4^). Across the sampling interval, we found no significant difference in pairwise IBD in ABS (*W* = 7,817,034, *p* = 0.89). Pairwise IBD decreased slightly from 2000 to 2008 in PLE, presumably driven either by a bump in immigration into this subpopulation during our sampling interval or by sampling stochasticity (Table 1; Wilcoxon rank‐sum test: *W* = 97,115, *p* = 8.27 × 10^−5^). This decrease explains the more comparable levels of pairwise IBD between ABS and PLE in 2008.

To better understand the degree of relatedness between subpopulations, we generated all possible draws of one ABS and one PLE bird and estimated the shared IBD of these inter‐subpopulation pairs in both 2000 and 2008. We then compared the results of these new analyses to our previous calculations of pairwise IBD among individuals within each respective subpopulation. In 2000, average between‐subpopulation IBD (ABSvPLE) was lower than values observed within each subpopulation (Table 1), although this difference was only statistically significant for PLE (Figure 4a; Wilcoxon rank‐sum test: *W* = 1,193,600, *p* = 0.08 for ABS and *W* = 369,640, *P* = 2.22 × 10^−5^ for PLE). A similar trend was also observed in 2008, although the comparison is now only significant for ABS (Wilcoxon rank‐sum test: *W* = 197,130,000, *p* = 2.26 × 10^−16^ for ABS, and *W* = 9,880,600, *p* = 0.31 for PLE). In general, birds across the two subpopulations were less related to each other than birds within each subpopulation.

Finally, we focused on only the closest pairwise relationships (those with shared IBD ≥0.09 indicating first cousins or greater), as these likely contribute the most to the degree of relatedness in each subpopulation. As expected, ABS had an overall smaller proportion of close relationships than PLE in both years (Figure 4b; Pearson's Chi‐squared test: *X* ^2^ = 9.89, *df* = 1, *P* = 1.6 × 10^−3^). Notably, PLE had a significantly higher proportion of relationship pairs within the high (parent–offspring and full sibling) and moderate (half sibling, avuncular, and grandparent–grandchild) relatedness classes than ABS in both years (Figure 4b; Pearson's Chi‐squared test: *X* ^2^ = 29.08, *df* = 1, *p* = 6.92 × 10^−8^). From 2000 to 2008, the overall proportions of close relationships did not significantly change in ABS (from 0.041 to 0.055; Pearson's Chi‐squared test: *X* ^2^ = 3.34, *df* = 1, *p* = 0.06) but decreased in PLE (from 0.115 to 0.063; Pearson's Chi‐squared test: *X* ^2^ = 8.05, *df* = 1, *p* = 4.6 × 10^−3^). This decrease in PLE is driven by a reduction in first cousin pairs across the sampling period (Figure 4b; from 0.07 to 0.02; Pearson's Chi‐squared test: *X* ^2^ = 17.56, *df* = 1, *P* = 2.79 × 10^−5^) and likely contributed to the overall lowered pairwise IBD in 2008 observed previously within this subpopulation.

## 4 DISCUSSION

We leveraged a unique opportunity to test empirically a prevailing conservation genetics paradigm within a metapopulation context, in a bird species known to be highly sensitive to human disturbance and residential development (Chen et al., 2016; Coulon et al., 2012). Our results reveal how quickly genomic impacts can accumulate during rapid population decline, even in the presence of ongoing gene flow. In just a few generations after the onset of residential development, the declining population (PLE) already exhibited lower site‐based heterozygosity and higher levels of inbreeding, ROH, and relatedness than did the intact population occupying native habitat located only a few kilometers away (ABS). That we detected no significant changes in heterozygosity or inbreeding in either subpopulation across an 8‐year sampling interval indicates that genetic differences had already arisen by the year 2000 between these recently continuous subpopulations. Despite a decrease in overall pairwise IBD in PLE across our sampling period, plausibly driven by sampling stochasticity or far dispersing immigrants from diverged populations that entered PLE within our 8‐year interval, PLE still had a significantly larger proportion of the high and moderate relatedness classes than ABS for either year, suggestive of ongoing inbreeding.

The relative length distributions of ROH provide further insight into the demographic histories of our subpopulations. ROH are useful estimators of inbreeding in natural systems because *F*_ROH_ is correlated with homozygous mutation load and their length distributions can be used to approximate levels of inbreeding depression across populations within a species (Kardos, Nietlisbach, & Hedrick, 2018b; Stoffel et al., 2021). Longer ROH are expected to harbor more deleterious alleles with larger average effects on fitness than shorter ROH where purifying selection has had more time to purge maladaptive mutations (Stoffel et al., 2021; Szpiech et al., 2013). ROH lengths can also reveal demographic histories of populations (Grossen et al., 2020; Kardos, Åkesson, et al., 2018a; Ralph & Coop, 2013). Consistent with expectations for a recently declining population, we found higher proportions of ROH ≥5 Mb in PLE 2008 than in ABS, indicative of recent inbreeding associated with habitat loss and fragmentation in this subpopulation. *F*_ROH_ was also higher in PLE for both sampling periods, possibly reflecting a risk of more severe inbreeding depression and mutational load in this subpopulation. Notably, we recorded similar proportions of shorter ROH <5 Mb between ABS and PLE, consistent with these being one continuous population of shared demographic history in the past that has now begun to diverge in levels of inbreeding with recent local population decline.

Given that PLE already displayed lower heterozygosity and elevated inbreeding than ABS by our sampling period in 2000, despite exhibiting a census size comparable to ABS in 1998 (Figure 1a), PLE likely was experiencing genetic consequences of population decline well before the process was evident in our field monitoring in 1990s. Suburban supplemental feeding during the 1990s helped maintain high observed counts of FSJs in PLE; however, if these individuals were unsuccessful at establishing territories in the fragmented environment and ultimately failed to breed, the effective population size would have been much smaller than the census population size estimated with count data. Furthermore, previous work already documented higher rates of egg hatching failure and fledgling mortality within PLE as compared to ABS during the 1990s (Bowman & Woolfenden, 2001). Finally, even a small degree of habitat fragmentation has been shown to drastically impede movement of FSJ within a subpopulation due to their limited dispersal (Coulon et al., 2012). This limited dispersal would reduce overall admixture and facilitate pockets of high inbreeding associated with local population structure. Together, these studies coupled with our genomic findings suggest that birds in PLE probably began losing heterozygosity and increasing inbreeding with the onset of suburbanization in the 1980s, a decade before we captured any signals of population decline in our demographic monitoring. Our study emphasizes the importance of understanding the genomic consequences of declining populations—even while they still appear to be robust—as a critical consideration for conservation management.

Although we know that ABS and PLE began as segments of one large, continuous population, we cannot eliminate the possibility that PLE already harbored less heterozygosity than ABS even before the onset of housing development in the 1980s. Analysis of additional historic samples could capture population dynamics from before the beginning of habitat loss, providing a more complete understanding of population demographics at that time. Meanwhile, our study documented the surprising speed at which habitat fragmentation can result in reduced genetic health of local subpopulations of FSJs. Our simulations also confirmed that the magnitude of changes in allele frequencies between the two subpopulations prior to the year 2000 were plausible given the magnitude of changes in population sizes and increased isolation between the two subpopulations (Figure S3). Although we were unable to detect very short ROH given the inherent sparsity of our SNP array, the relative length distributions of ROH for each subpopulation are still informative for contrasting inbreeding dynamics. Recently, we obtained high‐coverage whole‐genome sequences from ABS and PLE from before the population crash, data which will help gain a more complete understanding of the genomic trajectories within these subpopulations in future studies.

Recent studies focused on population decline in other populations, such as in the Channel Island fox and Alpine ibex (Grossen et al., 2020; Robinson et al., 2016, 2018), underscore the role of past demographics in either accumulation of deleterious variation or purging of that variation from the genome. These studies suggest populations with repeated bottlenecks in the past may have purged deleterious recessive variation to the point that they are much less susceptible to inbreeding depression, leading some to emphasize the importance of quantifying deleterious variation for conservation purposes (Teixeira & Huber, 2021). However, populations that have not faced recurrent bottlenecks are expected to show a strong correlation between reduced genome‐wide diversity and increased inbreeding depression, retaining the maintenance of genetic diversity as a useful strategy for conservation (Kardos et al., 2021; Ralls et al., 2020). Reduced genetic diversity has been associated with reduced egg hatchability and juvenile survival in the FSJ (Chen et al., 2016), establishing the utility of metrics of genome‐wide genetic diversity in this system. Therefore, in this case, neutral variation likely serves as a useful proxy of incipient genetic risks imposed by loss of heterozygosity, especially in the context of comparing formerly connected subpopulations that are becoming increasingly fragmented and depopulated.

Our results highlight how quickly genomic impacts can accumulate during rapid anthropogenic population decline, even in the presence of ongoing gene flow. Habitat loss and fragmentation have been shown to deteriorate genetic diversity at the greatest magnitude relative to other modes of range contraction (Rogan et al., 2021). Therefore, we emphasize the importance of conservation intervention in the early stages of population decline, especially given the severity and speed associated with habitat loss and fragmentation. Furthermore, gene flow from even depauperate and inbred subpopulations (like PLE) are critical for the persistence of seemingly stable, large subpopulations (like ABS), especially in the FSJ (Chen et al., 2016). We underscore the importance of maintaining these small subpopulations and increasing connectivity between contiguous subpopulations for vulnerable species in a metapopulation context. Our study exemplifies how consideration of all aspects of a population's genetic attributes, from immigration to the proportion of closely related individuals, should be evaluated for a comprehensive understanding of the genomic dynamics for conservation decisions.

## CONFLICT OF INTEREST

The authors declare that they have no competing interests.

## Supporting information


Appendix S1

## Data Availability

Genetic data for ABS and PLE and R code for the Wright‐Fisher simulation are available at https://doi.org/10.6084/m9.figshare.13507440.v1.

## References

[eva13421-bib-0001] Aguillon, S. M. , Fitzpatrick, J. W. , Bowman, R. , Schoech, S. J. , Clark, A. G. , Coop, G. , & Chen, N. (2017). Deconstructing isolation‐by‐distance: The genomic consequences of limited dispersal. PLoS Genetics, 13, e1006911. 28771477 10.1371/journal.pgen.1006911 PMC5542401

[eva13421-bib-0002] Anderson, R. M. , & May, R. M. (1986). The invasion, persistence and spread of infectious diseases within animal and plant communities. Philosophical Transactions of the Royal Society B: Biological Sciences, 314, 533–570. 10.1098/rstb.1986.0072 2880354

[eva13421-bib-0003] Barrett, R. , & Schluter, D. (2008). Adaptation from standing genetic variation. Trends in Ecology & Evolution, 23, 38–44. 18006185 10.1016/j.tree.2007.09.008

[eva13421-bib-0004] Bosse, M. , Megens, H.‐J. , Madsen, O. , Paudel, Y. , Frantz, L. A. F. , Schook, L. B. , Crooijmans, R. P. M. A. , & Groenen, M. A. M. (2012). Regions of homozygosity in the porcine genome: Consequence of demography and the recombination landscape. PLoS Genetics, 8, e1003100. 23209444 10.1371/journal.pgen.1003100 PMC3510040

[eva13421-bib-0005] Boughton, R.K. , and Bowman, R. (2011). State wide assessment of Florida Scrub‐jays on managed areas: A comparison of current populations to the results of the 1992‐93 survey. 43.

[eva13421-bib-0006] Bowman, R. , & Woolfenden, G. E. (2001). Nest success and the timing of nest failure of Florida Scrub‐jays in suburban and wildland habitats. In J. M. Marzluff , R. Bowman , & R. Donnelly (Eds.), Avian ecology and conservation in an urbanizing world (pp. 383–402). Springer US.

[eva13421-bib-0007] Brooks, T. M. , Mittermeier, R. A. , Mittermeier, C. G. , Fonseca, G. A. B. D. , Rylands, A. B. , Konstant, W. R. , Flick, P. , Pilgrim, J. , Oldfield, S. , Magin, G. , et al. (2002). Habitat loss and extinction in the hotspots of biodiversity. Conservation Biology, 16, 909–923.

[eva13421-bib-0008] Charlesworth, B. (2009). Fundamental concepts in genetics: Effective population size and patterns of molecular evolution and variation. Nature Reviews Genetics, 10, 195–205. 10.1038/nrg2526 19204717

[eva13421-bib-0009] Charlesworth, D. , & Charlesworth, B. (1987). Inbreeding depression and its evolutionary consequences. Annual Review of Ecology and Systematics, 18, 237–268.

[eva13421-bib-0010] Chen, N. , Hout, C. V. V. , Gottipati, S. , & Clark, A. G. (2014). Using Mendelian inheritance to improve high throughput SNP discovery. Genetics, 198, 847–857. 25194160 10.1534/genetics.114.169052 PMC4224174

[eva13421-bib-0011] Chen, N. , Cosgrove, E. J. , Bowman, R. , Fitzpatrick, J. W. , & Clark, A. G. (2016). Genomic consequences of population decline in the endangered Florida Scrub‐jay. Current Biology, 26, 2974–2979. 27746026 10.1016/j.cub.2016.08.062 PMC5102777

[eva13421-bib-0012] Coulon, A. , Fitzpatrick, J. W. , Bowman, R. , Stith, B. M. , Makarewich, C. A. , Stenzler, L. M. , & Lovette, I. J. (2008). Congruent population structure inferred from dispersal behaviour and intensive genetic surveys of the threatened Florida scrub‐jay (*Aphelocoma cœrulescens*). Molecular Ecology, 17, 1685–1701. 18371014 10.1111/j.1365-294X.2008.03705.x

[eva13421-bib-0013] Coulon, A. , Fitzpatrick, J. W. , Bowman, R. , & Lovette, I. J. (2012). Mind the gap: Genetic distance increases with habitat gap size in Florida scrub jays. Biology Letters, 8, 582–585. 22357936 10.1098/rsbl.2011.1244 PMC3391449

[eva13421-bib-0014] Frankham, R. (2005). Genetics and extinction. Biological Conservation, 126, 131–140.

[eva13421-bib-0015] Franklin, I. R. (1977). The distribution of the proportion of the genome which is homozygous by descent in inbred individuals. Theoretical Population Biology, 11, 60–80. 404725 10.1016/0040-5809(77)90007-7

[eva13421-bib-0016] Gao, L.‐Z. , & Gao, C.‐W. (2016). Lowered diversity and increased inbreeding depression within peripheral populations of wild Rice *Oryza rufipogon* . PLoS One, 11, e0150468. 26963913 10.1371/journal.pone.0150468 PMC4786333

[eva13421-bib-0017] Groom, M. J. , Meffe, G. K. , & Carroll, R. C. (2012). Principles of Conservation Biology. Sinauer Associates, Inc.

[eva13421-bib-0018] Grossen, C. , Guillaume, F. , Keller, L. F. , & Croll, D. (2020). Purging of highly deleterious mutations through severe bottlenecks in alpine ibex. Nature Communications, 11, 1001. 10.1038/s41467-020-14803-1 PMC7035315 32081890

[eva13421-bib-0019] Hanski, I. (2005). The shrinking world: Ecological consequences of habitat loss. International Ecology Institute.

[eva13421-bib-0020] Hanski, I. (2011). Habitat loss, the dynamics of biodiversity, and a perspective on conservation. Ambio, 40, 248–255. 21644453 10.1007/s13280-011-0147-3 PMC3357798

[eva13421-bib-0021] Hedrick, P. W. (2001). Conservation genetics: Where are we now? Trends in Ecology & Evolution, 16, 629–636.

[eva13421-bib-0022] Huisman, J. , Kruuk, L. E. B. , Ellis, P. A. , Clutton‐Brock, T. , & Pemberton, J. M. (2016). Inbreeding depression across the lifespan in a wild mammal population. Proceedings of the National Academy of Sciences of the United States of America, 113, 3585–3590. 26979959 10.1073/pnas.1518046113 PMC4822623

[eva13421-bib-0023] Humble, E. , Paijmans, A. J. , Forcada, J. , & Hoffman, J. I. (2020). An 85K SNP array uncovers inbreeding and cryptic relatedness in an antarctic fur seal breeding colony. G3 (Bethesda), 10, 2787–2799. 32540866 10.1534/g3.120.401268 PMC7407454

[eva13421-bib-0024] Kardos, M. , Åkesson, M. , Fountain, T. , Flagstad, Ø. , Liberg, O. , Olason, P. , Sand, H. , Wabakken, P. , Wikenros, C. , & Ellegren, H. (2018a). Genomic consequences of intensive inbreeding in an isolated wolf population. Nature Ecology & Evolution, 2, 124–131. 29158554 10.1038/s41559-017-0375-4

[eva13421-bib-0025] Kardos, M. , Nietlisbach, P. , & Hedrick, P. W. (2018b). How should we compare different genomic estimates of the strength of inbreeding depression? Proceedings of the National Academy of Sciences of the United States of America, 115, E2492–E2493. 29467294 10.1073/pnas.1714475115 PMC5856524

[eva13421-bib-0026] Kardos, M. , Armstrong, E. E. , Fitzpatrick, S. W. , Hauser, S. , Hedrick, P. W. , Miller, J. M. , Tallmon, D. A. , & Funk, W. C. (2021). The crucial role of genome‐wide genetic variation in conservation. Proceedings of the National Academy of Sciences of the United States of America, 118, e2104642118. 34772759 10.1073/pnas.2104642118 PMC8640931

[eva13421-bib-0027] Kawamura, K. (2005). Low genetic variation and inbreeding depression in small isolated populations of the Japanese rosy bitterling, *Rhodeus ocellatus* kurumeus. Zoological Science, 22, 517–524. 15930824 10.2108/zsj.22.517

[eva13421-bib-0028] Kohn, M. H. , Murphy, W. J. , Ostrander, E. A. , & Wayne, R. K. (2006). Genomics and conservation genetics. Trends in Ecology & Evolution, 21, 629–637. 16908089 10.1016/j.tree.2006.08.001

[eva13421-bib-0029] Liberg, O. , Andrén, H. , Pedersen, H.‐C. , Sand, H. , Sejberg, D. , Wabakken, P. , Åkesson, M. , & Bensch, S. (2005). Severe inbreeding depression in a wild wolf (*Canis lupus*) population. Biology Letters, 1, 17–20. 17148117 10.1098/rsbl.2004.0266 PMC1629062

[eva13421-bib-0030] Lynch, M. , Conery, J. , & Burger, R. (1995). Mutation accumulation and the extinction of small populations. American Naturalist, 146, 489–518.

[eva13421-bib-0031] Meyermans, R. , Gorssen, W. , Buys, N. , & Janssens, S. (2020). How to study runs of homozygosity using PLINK? A guide for analyzing medium density SNP data in livestock and pet species. BMC Genomics, 21, 1–14. 10.1186/s12864-020-6463-x PMC6990544 31996125

[eva13421-bib-0032] Narasimhan, V. , Danecek, P. , Scally, A. , Xue, Y. , Tyler‐Smith, C. , & Durbin, R. (2016). BCFtools/RoH: A hidden Markov model approach for detecting autozygosity from next‐generation sequencing data. Bioinformatics, 32, 1749–1751. 26826718 10.1093/bioinformatics/btw044 PMC4892413

[eva13421-bib-0033] O'Connell, J. R. , & Weeks, D. E. (1998). PedCheck: A program for identification of genotype incompatibilities in linkage analysis. American Journal of Human Genetics, 63, 259–266. 9634505 10.1086/301904 PMC1377228

[eva13421-bib-0034] Ouborg, N. J. , Vergeer, P. , & Mix, C. (2006). The rough edges of the conservation genetics paradigm for plants. Journal of Ecology, 94, 1233–1248.

[eva13421-bib-0035] Pearman, P. B. , & Garner, T. W. J. (2005). Susceptibility of Italian agile frog populations to an emerging strain of Ranavirus parallels population genetic diversity. Ecology Letters, 8, 401–408.

[eva13421-bib-0036] Pemberton, T. J. , Absher, D. , Feldman, M. W. , Myers, R. M. , Rosenberg, N. A. , & Li, J. Z. (2012). Genomic patterns of homozygosity in worldwide human populations. American Journal of Human Genetics, 91, 275–292. 22883143 10.1016/j.ajhg.2012.06.014 PMC3415543

[eva13421-bib-0037] Purcell, S. , Neale, B. , Todd‐Brown, K. , Thomas, L. , Ferreira, M. A. R. , Bender, D. , Maller, J. , Sklar, P. , de Bakker, P. I. W. , Daly, M. J. , & Sham, P. C. (2007). PLINK: A tool set for whole‐genome association and population‐based linkage analyses. American Journal of Human Genetics, 81, 559–575. 17701901 10.1086/519795 PMC1950838

[eva13421-bib-0038] R Core Team . (2015). R: A language and environment for statistical computing. R Foundation for Statistical Computing.

[eva13421-bib-0039] Ralls, K. , Sunnucks, P. , Lacy, R. C. , & Frankham, R. (2020). Genetic rescue: A critique of the evidence supports maximizing genetic diversity rather than minimizing the introduction of putatively harmful genetic variation. Biological Conservation, 251, 108784.

[eva13421-bib-0040] Ralph, P. , & Coop, G. (2013). The geography of recent genetic ancestry across Europe. PLoS Biology, 11, e1001555. 23667324 10.1371/journal.pbio.1001555 PMC3646727

[eva13421-bib-0041] Robinson, J. A. , Ortega‐Del Vecchyo, D. , Fan, Z. , Kim, B. Y. , vonHoldt, B. M. , Marsden, C. D. , Lohmueller, K. E. , & Wayne, R. K. (2016). Genomic flatlining in the Endangered Island fox. Current Biology, 26, 1183–1189. 27112291 10.1016/j.cub.2016.02.062

[eva13421-bib-0042] Robinson, J. A. , Brown, C. , Kim, B. Y. , Lohmueller, K. E. , & Wayne, R. K. (2018). Purging of strongly deleterious mutations explains long‐term persistence and absence of inbreeding depression in Island foxes. Current Biology, 28, 3487–3494.e4. 30415705 10.1016/j.cub.2018.08.066 PMC6462144

[eva13421-bib-0043] Rogan, J. , Parker, M.R. , Hancock, Z.B. , Earl, A.D. , Buchholtz, E.K. , Chyn, K. , Martina, J. , and Fitzgerald, L.A. (2021). Paths to annihilation: Genetic and demographic consequences of range contraction patterns. BioRxiv 2021.01.26.428313. 10.1038/s41598-023-28927-z PMC9886963 36717685

[eva13421-bib-0044] Soulé, M. E. , & Simberloff, D. (1986). What do genetics and ecology tell us about the design of nature reserves? Biological Conservation, 35, 19–40.

[eva13421-bib-0045] Spielman, D. , Brook, B. W. , Briscoe, D. A. , & Frankham, R. (2004). Does inbreeding and loss of genetic diversity decrease disease resistance? Conservation Genetics, 5, 439–448.

[eva13421-bib-0046] Stith, B. M. , Fitzpatrick, J. W. , Woolfenden, G. E. , & Pranty, B. (1996). Classification and conservation of metapopulations: A case study of the Florida Scrub jay. In D. R. McCullough (Ed.), Metapopulations and Wildlife Conservation (pp. 187–216). Island Press.

[eva13421-bib-0047] Stoffel, M. A. , Johnston, S. E. , Pilkington, J. G. , & Pemberton, J. M. (2021). Mutation load decreases with haplotype age in wild soay sheep. Evolution Letters, 5, 187–195. 34136268 10.1002/evl3.229 PMC8190445

[eva13421-bib-0048] Suh, Y. H. , Pesendorfer, M. B. , Tringali, A. , Bowman, R. , & Fitzpatrick, J. W. (2020). Investigating social and environmental predictors of natal dispersal in a cooperative breeding bird. Behavioral Ecology, 31, 692–701.

[eva13421-bib-0049] Szpiech, Z. A. , Xu, J. , Pemberton, T. J. , Peng, W. , Zöllner, S. , Rosenberg, N. A. , & Li, J. Z. (2013). Long runs of homozygosity are enriched for deleterious variation. American Journal of Human Genetics, 93, 90–102. 23746547 10.1016/j.ajhg.2013.05.003 PMC3710769

[eva13421-bib-0050] Tarpy, D. R. (2003). Genetic diversity within honeybee colonies prevents severe infections and promotes colony growth. Proceedings of the Royal Society B: Biological Sciences, 270, 99–103. 10.1098/rspb.2002.2199 PMC1691209 12596763

[eva13421-bib-0051] Teixeira, J. C. , & Huber, C. D. (2021). The inflated significance of neutral genetic diversity in conservation genetics. Proceedings of the National Academy of Sciences, 118, e2015096118. 10.1073/pnas.2015096118 PMC7958437 33608481

[eva13421-bib-0052] Thompson, E. A. (2013). Identity by descent: Variation in meiosis, across genomes, and in populations. Genetics, 194, 301–326. 23733848 10.1534/genetics.112.148825 PMC3664843

[eva13421-bib-0053] Whiteman, N. K. , Matson, K. D. , Bollmer, J. L. , & Parker, P. G. (2006). Disease ecology in the Galapagos hawk (*Buteo galapagoensis*): Host genetic diversity, parasite load and natural antibodies. Proceedings of the Royal Society B: Biological Sciences, 273, 797–804. 10.1098/rspb.2005.3396 PMC1560217 16618672

[eva13421-bib-0054] Wickham, H. (2009). ggplot2: Elegant graphics for data analysis. Springer‐Verlag.

[eva13421-bib-0055] Woolfenden, G. , and Fitzpatrick, J.W. (1984). The Florida Scrub jay: Demography of a cooperative‐breeding bird. Princeton University Press 20.

[eva13421-bib-0056] Yang, J. , Lee, S. H. , Goddard, M. E. , & Visscher, P. M. (2011). GCTA: A tool for genome‐wide complex trait analysis. American Journal of Human Genetics, 88, 76–82. 21167468 10.1016/j.ajhg.2010.11.011 PMC3014363

[eva13421-bib-0057] Zheng, X. , Levine, D. , Shen, J. , Gogarten, S. M. , Laurie, C. , & Weir, B. S. (2012). A high‐performance computing toolset for relatedness and principal component analysis of SNP data. Bioinformatics, 28, 3326–3328. 23060615 10.1093/bioinformatics/bts606 PMC3519454

