# Supplementary material for: Dynamics of reduced genetic diversity in increasingly fragmented populations of Florida scrub jays,Aphelocoma coerulescens
Source: Evol Appl. 2022 Jun 1;15(6):1018–27. doi: 10.1111/eva.13421 (PMC9234620; doi:10.1111/eva.13421)
Supplement: Supplementary file 1 — Appendix S1 [file EVA-15-1018-s001.docx]

**Supplemental Information for:**

**Dynamics of reduced genetic diversity in increasingly fragmented populations of Florida Scrub-Jays, *Aphelocoma coerulescens***

| **Figure S1.** Subpopulation structure analysis | Page 2 |
| --- | --- |
| **Figure S2.**  Observed change in allele frequencies | Page 3 |
| **Figure S3.**  Goodness-of-fit of Wright-Fisher simulation of the observed changes in allele frequencies | Page 4 |
| **Figure S4.**  SNP distribution across the genome | Page 5 |
| **Figure S5.**  Distribution of ROH segments in subsamples of ABS | Page 6 |
| **Figure S6.** Correlation between F_ROH_ and F^III^ | Page 7 |
| **Figure S7.**  Distribution of ROH segments across the genome | Page 8 |
| **Supplemental Methods Procedures** | Page 9 |
| **Supplemental References** | Page 12 |

**Subpopulation structure analysis**

**(A) (B)**

**Figure S1.** (A) Principal components analysis (PCA) using samples from both subpopulations. ABS samples display a wider spread, indicative of greater variation, as expected in a larger, stable subpopulation. The single PLE 2008 individual clustering with ABS 2008 (indicated with a red arrow) represents a known immigrant from ABS into PLE. (B) Separate PCAs of ABS and PLE do not detect cryptic substructure within each subpopulation.

**Observed change in allele frequencies from 2000 to 2008**

**
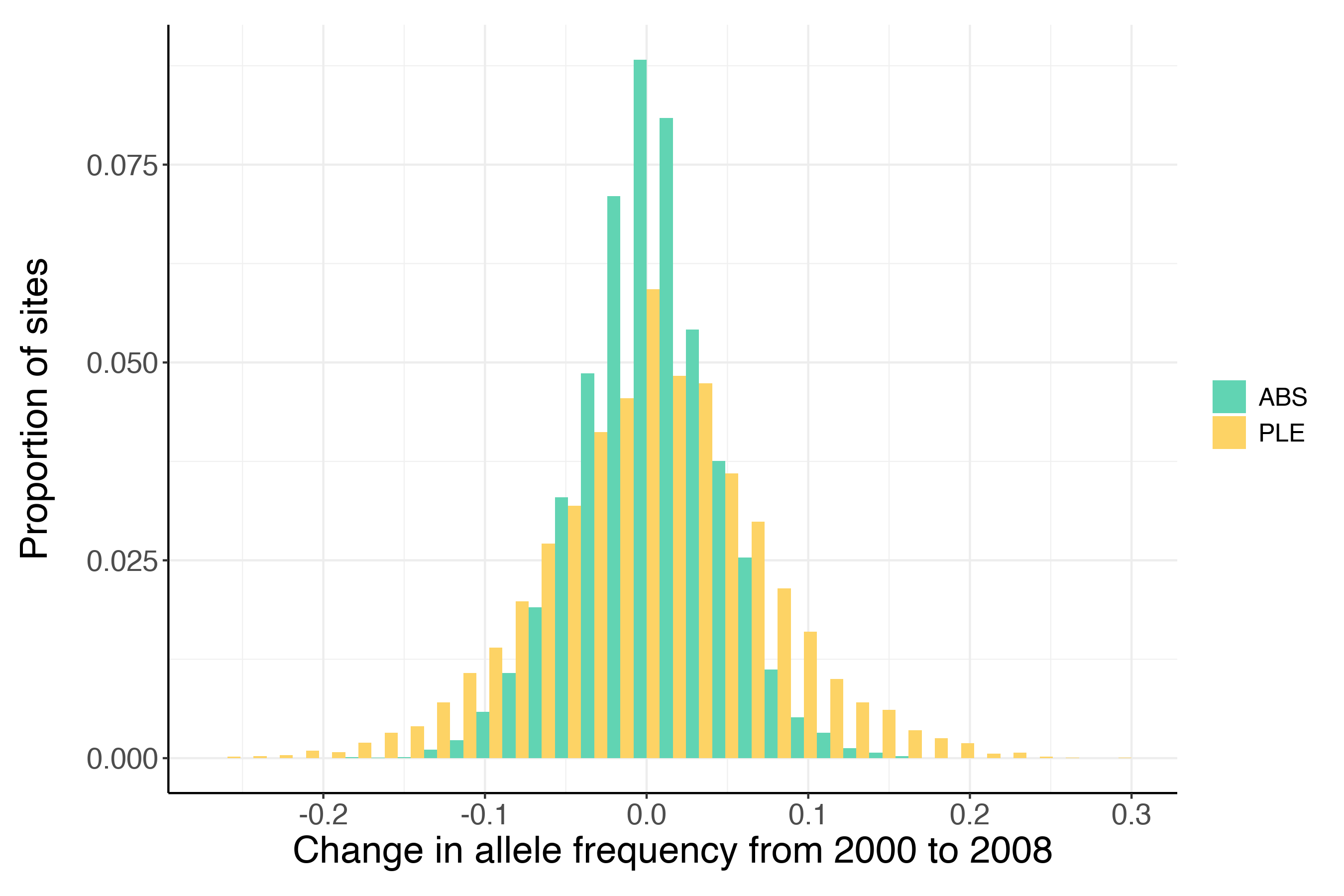
**

**Figure S2.** Histogram of the observed changes in allele frequency in ABS and PLE from 2000 to 2008 (Δ*p*). Mean Δ*p* for the populations are 0.002 and 0.003, respectively. As expected in a declining population of small effect size, the variance in Δ*p* in PLE (0.005) is much higher than in ABS (0.002).

**Goodness-of-fit of Wright-Fisher simulation of the observed changes in allele frequencies**


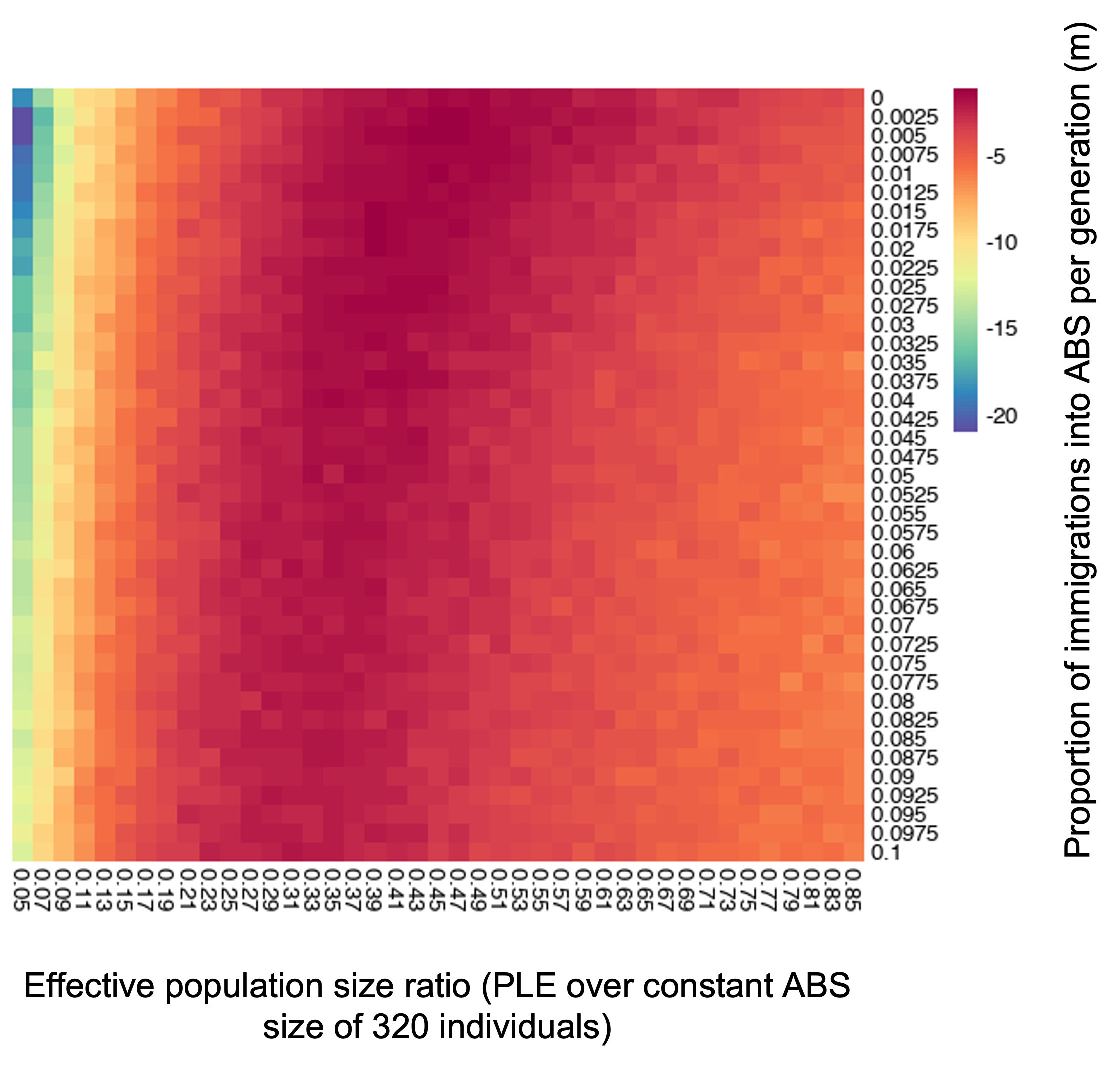


**Figure S3.** -Log_10_(*P*-value) surface plot showing the goodness-of fit of the observed changes in allele frequencies at 7,834 SNPs to a Wright-Fisher-with-migration simulation (described in the Supplemental Methods). Briefly, we simulated 4 generations forward-in-time for each population (8 generations of divergence), with the ABS population as the founder (as though the ABS and PLE populations were one panmictic unit in 1980) followed by a single sharp reduction in *N_PLE_* size ratio (the effective population size of PLE over a constant population size of 320 individuals in ABS) along with reduced migration. Plot values are based on Kolmogorov-Smirnov tests for similarity of distributions of allele frequency changes across 50 simulation replicates. The cooler colors on the plot indicate a poor fit to the data and can be considered parameter sets that can be rejected. The likelihood surface has a ridge where a smaller effective size ratio with relatively higher migration fits about as well as a larger effective size ratio with less migration.

**SNP distribution across the genome**

**Figure S4.** Boxplot of distances between adjacent SNPs for each autosome (numbered according to homology with the Zebra Finch genome). The bottom, bolded middle, topmost lines of the boxes represent the 1^st^ quartile, mean, and 3^rd^ quartile, respectively. Only 10 out of 11,737 autosomal SNPs had adjacent distances greater than 300 Kb and were excluded from the plot for visualization purposes. Across the genome, the average distance between adjacent SNPs was 85.5 Kb. SNP density per chromosome was approximately 300 SNPs. Our markers covered 32 of 39 known homologous autosomes, excluding chromosome 16 and many microchromosomes.

**Distribution of ROH counts from a subsample of ABS birds**

**
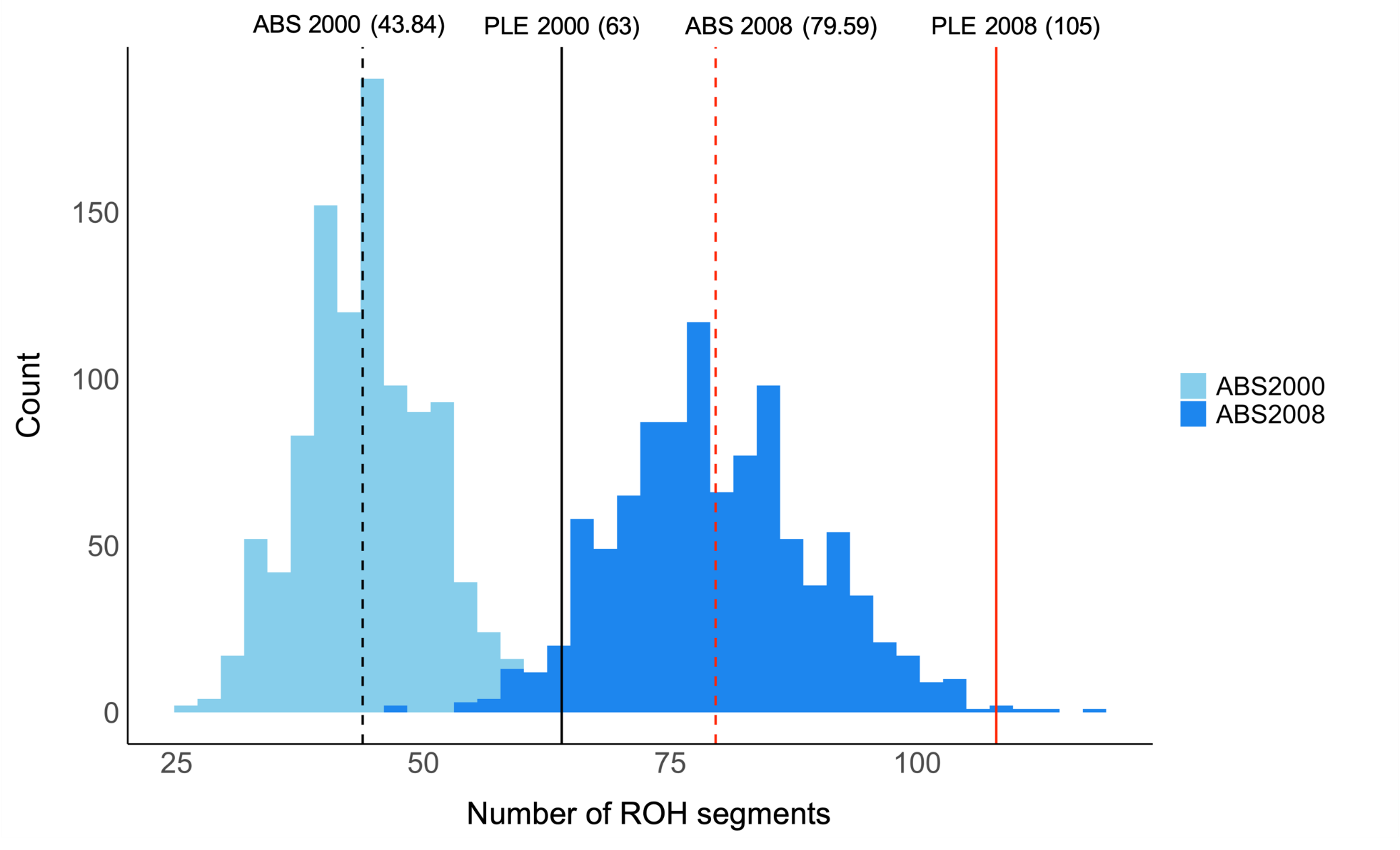
**

**Figure S5.** Because the number of detectable ROH segments scales with sample size, we account for the disparity in sample sizes between ABS and the suburban PLE by randomly subsampling ABS 1000 times (N = 24 in 2000, N = 41 in 2008) to match our PLE sample sizes. The distributions of the lengths of ROH for each ABS subsample are plotted here and the mean number of segments are denoted with dashed lines (years 2000 and 2008 represented with black and red, respectively). Observed PLE estimates (solid lines) are then compared to the mean of each ABS distribution using a Wilcoxon rank-sum test. The mean of our subsampled ABS distribution was 43.84 ± 0.19 for 2000 and 79.59 ± 0.32 in 2008, which is consistently lower than the number of ROH detected in PLE for either year (Wilcoxon rank sum test: *W* = 0, *P* = 0.041 in year 2000 and *W* = 4.5, *P* = 0.043 in year 2008).

**Correlation between inbreeding coefficients *F_ROH_* and *F^III^***

**
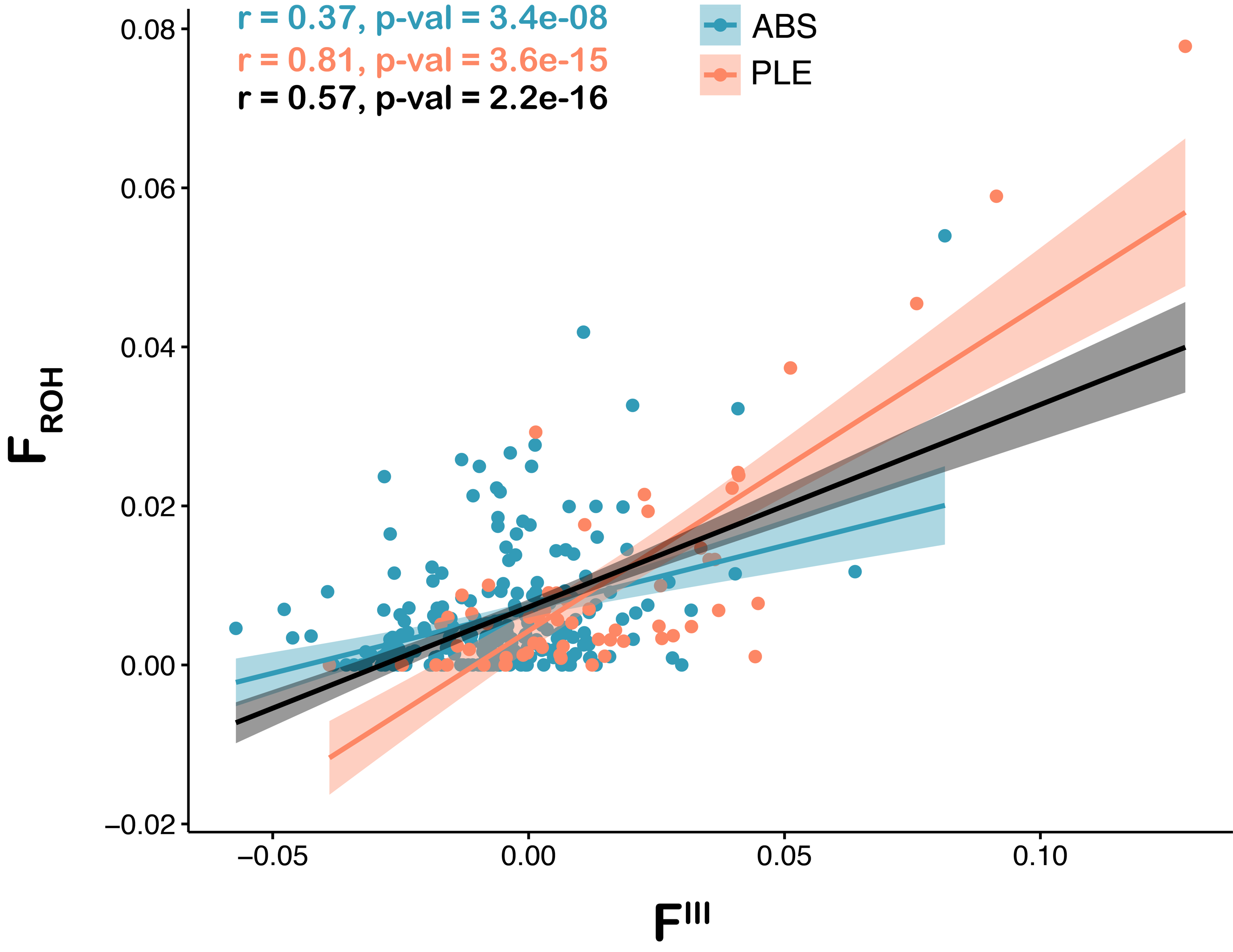
**

**Figure S6.** Relationship between inbreeding coefficients *F_ROH_* and *F^III^* for ABS and PLE pooled across 2000 and 2008. Pearson correlation and *P*-values derived from Pearson’s correlation tests for ABS, PLE, and the entire dataset is denoted in blue, orange, and black, respectively. The Pearson correlation for PLE (*r* = 0.81) is higher than for ABS (*r* = 0.37) because our SNP density captures only recent inbreeding (ROH with length ≥ 1 Mb). Thus, the tighter correlation between *F_ROH_* and *F^III^* reflects this more recent inbreeding in PLE than ABS.

**Distribution of ROH segments across the genome**

**Figure S7.** Count of ROH segments detected across the genome. In both subpopulations, we found a total of 577 ROH on 28 of the 32 autosomes in which we had SNP markers available. Given our SNP sparsity, it is possible that there were also ROHs <1 Mb that we were unable to detect. However, the relative proportions of ROH segments between the subpopulations remain informative.

**Supplemental Methods Procedures**

**Runs of Homozygosity detection**

To identify ROH, we used a sliding window approach in PLINK with the --homozyg option on a dataset of 11,737 autosomal SNPs. We removed any SNPs that diverged significantly from Hardy-Weinberg equilibrium by imposing a *P-*value threshold of 0.001. We did not do linkage disequilibrium pruning as it often reduces detection of ROH and thus biases estimates of *F_ROH_* (Meyermans, Gorssen, Buys, & Janssens, 2020). We set the scanning window size at 50 SNPs (--homozyg-window-snp) and required that no more than 1 heterozygous call and 5 missing calls were allowed in each ROH. We only considered distinct ROH segments with gap sizes >1000 kb (--homozyg-gap). To account for the low density of our SNP markers along our genome (average distance between adjacent SNPs ~ 85.5 kb; Fig. S2), we required ROH to contain at least 8 SNPs (--homozyg-snp), have a minimum length of 1 Mb (homozyg-kb), and to have at least one SNP per 200 kb on average (--homozyg-density). To calculate *F_ROH_*, we summed the lengths of all ROH segments within an individual outputted in our PLINK analysis as a proportion out of 1.2 Gb, the genome size of the closely-related American Crow, *Corvus brachyrhyncho* (Kapusta, Suh, & Feschotte, 2017*)*.

**Detection of ROH using BCFtools/RoH**

In addition to PLINK, we detected ROH using BCFtools/RoH (Narasimhan et al., 2016) and compared the results across the programs. PLINK implements a genotype-counting method to search for consecutive homozygous genotypes and allows for user-defined thresholds for the maximum numbers of heterozygotes and missing genotypes (Purcell et al., 2007; Szpiech, Blant, & Pemberton, 2017). BCFtools/RoH utilizes a hidden Markov model to discriminate between autozygous and non-autozygous regions while incorporating information on allele frequency (Narasimhan et al., 2016). We ran BCFtools/RoH (option -G30) on the same dataset of 11,737 autosomal SNPs without LD pruning and read in allele frequencies using the command --AF-file. Finally, we retained only ROH segments with high posterior quality scores of ≥25.

BCFtools/RoH detected a total of 651 ROH segments (compared to 577 segments using PLINK) on 31 chromosomes – including those found in our PLINK investigation plus 3 additional chromosomes (chromosome 22, 26, and 30). Specifically, BCFtool/RoH detected 103 and 66 ROH segments in ABS and PLE in the year 2000, respectively. In 2008, this increased to 381 and 101 segments in ABS and PLE. Despite the higher counts of total ROH segments detected with BCFtools/RoH, the relative proportions of ROH segments across the subpopulations and across our sampling points remained comparable between the programs, with more than 86% of PLINK ROH segments overlapping with segments found with BCFtools/RoH.

Trends in ROH abundance and distribution of lengths remained the same across the two programs. As with our PLINK analysis, we subsampled individuals in ABS to match our smaller PLE sample sizes and found that the mean number of ROH segments in our subsample of ABS was consistently lower than in PLE for both years (Wilcoxon rank sum test: *W* = 15, *P* = 0.046 in year 2000 and *W* = 16, *P* = 0.048 in year 2008). In addition, *F_ROH_* also remained significantly lower in ABS than in PLE across both years (Wilcoxon rank-sum test: *W* = 24510, *P* = 0.028).

Next, we compared the proportion of short (<5 Mb) versus long (≥5 Mb) ROH within ABS and PLE in each year and across both years pooled. Consistent with our PLINK results and predictions for a continuously declining subpopulation in PLE, we found that ABS had lower proportions of long ROH in each year. Specifically, these proportions were 0.19 in ABS and 0.50 in PLE for the year 2000 (Pearson’s Chi-square test: *X^2^* *=* 6.42, *df* = 1, *P* = 0.01), and 0.27 compared to 0.51 for 2008 (Pearson’s Chi-square test: *X^2^* *=* 6.43, *df* = 1, *P* = 0.01). This relationship also holds when we pooled the samples across years (Pearson’s Chi-square test: *X^2^* *=* 11.71, *df* = 1, *P* = 6.23 x 10^-4^). BCFtools/RoH detected both shorter and longer ROH than PLINK, however, mean ROH length was similar across the programs. ROH length ranged from approximately 1 Mb to 25 Mb for PLINK (mean 3.9 Mb ± 142.5 Kb) and from 253 Kb to 69.5 Mb (mean 4 Mb ± 234.4 Kb) for BCFtools/RoH. Notably, both programs recorded the longest ROH segment in PLE 2008.

**Pairwise IBD and identification of relationship types**

Relatedness was assessed with pairwise IBD, estimated in PLINK (“PI_HAT” value generated by --genome) both within and between subpopulations (ABSvPLE; Purcell et al., 2007). Specifically, we compared the mean proportion of the genome in IBD for each subpopulation (Figure 4A). We further investigated the closest pairwise relationships—those with IBD values ≥ 0.09 (Figure 4B). We defined pairwise IBD bins for each relationship class based on the range of observed pairwise IBD values for known relationships in the pedigree, with the minimum considered pairwise IBD of 0.09 corresponding to the lower end of observed pairwise IBD for known first-cousins. The resulting IBD bins for the relationship classes are as follows: High relatedness: (IBD ≥ 0.40) includes parent-offspring and full-sibling pairs, Moderate relatedness: (IBD ≥ 0.18-0.40) includes grandparent-grandchild, avuncular, and half-sibling pairs, and Other Close relatedness: (IBD ≥ 0.09-0.18) which includes first-cousin pairs. We quantified the proportion of relationships within these classes across all pairs for each subpopulation in both sampling periods.

**Generation time calculations**

We estimated generation time using ABS demography data from 1988 through 2016. We restricted the data set to observations of nestlings born in 1989 through 2015 and banded at ABS. We then filtered this dataset to include only observations for which the mother met the following criteria: age known, born in 1988 or later and not observed after 2015, and observed every year of her lifespan. The resulting dataset included 1,575 nestling observations. Generation time was estimated as the mean age of the female breeder across observations and it yielded a generation time estimate of 5.11 years.

We also estimated generation time using the demography data available for the PLE subpopulation. We restricted the data set to observations of nestlings born in 1993 through 2009 and banded at PLE, and again filtered based on female breeders meeting these criteria: age known, born in 1992 or later and not observed after 2009, and observed every year of her lifespan. This data set included 531 nestling observations and the generation time estimate for this dataset was 4.66 years.

**Assessing the plausibility of population parameters with simulations**

We ran simulations to provide a rough test of whether the reduction in genetic diversity observed in the SNP array data from the PLE population is consistent with population genetic expectations given plausible estimates of the change in population size and reduction in gene exchange with the ABS population. We conducted simulations for a pair of hypothetical populations, each undergoing classical Wright-Fisher neutral drift (recurrent binomial sampling) with unidirectional migration from PLE to ABS (in keeping with field documentation of this asymmetry based on banded jays). The simulations were initialized with the observed SNP allele frequencies in the ABS population for each of the 7,834 biallelic SNPs. Both populations were initialized in this way, as we assume panmixia at the start. Based on aerial photos, land use maps, and microsatellite data (Coulon et al., 2008), we know that the ABS and PLE populations were subsets of a larger, continuous, essentially panmictic population as recently as 1980, when residential development in PLE was just beginning, or about 4 generations before the 2000 census. While we know that habitat separation between the two populations gradually increased, for simplicity we assume that a single event produced a step difference in the PLE population size (with ABS remaining essentially unchanged in size).

The essential parameters we simulated include the two effective population sizes for PLE and ABS as well as the rate of migration between them. No successful breeder in PLE since 1990 was a migrant from ABS, but PLE migrants into ABS have successfully bred, so the only gene flow we consider is in the PLE-to-ABS direction. After migration each generation, the allele frequency for each SNP in ABS is (1-*m*) *p_ABS_* + *m p_PLE_*, where *p_PLE_* and *p_ABS_* are the pre-migration minor allele frequencies in PLE and ABS respectively. Because we are only interested in single-SNP statistics for these simulations, linkage is ignored. This sampling and migration process is continued for each of the 7,834 SNPs independently for 4 discrete generations. We fix *N_ABS_*, the size of the ABS population at 320, obtained from empirical census counts, and consider a range of sizes of the PLE population, allowing the ratio of effective sizes (*N_PLE_*/*N_ABS_*) to range from 0.05 to 0.85. We also consider a range of migration rates from 0 to 0.1. Both parameter ranges are broken into 40 equal intervals. Using each fixed value for migration and effective population size along the parameter range, we model neutral changes in allele frequencies across the 4 generations. For each parameter combination, the simulation generates a vector of minor allele frequencies for each population, and from these vectors we calculate the change in allele frequency at each SNP.

Figure S7 shows the average over 50 replicates of the probability surface as a contour heatmap and indicates the results of Kolmogorov-Smirnov tests for similarity of distributions of changes in allele frequencies. The darkest red color represents a failure to reject the null hypothesis of equivalent distributions of changes in allele frequencies. A large range of migration rates and effective sizes provide this fit, forming a band from the lower left and moving up and to the right. For lower estimates of migration rate, the effective size of PLE must be larger to obtain the best fit. The cooler colors on the plot indicate a poor fit to the data and can be considered parameter sets that can be rejected. Scanning over the full range of parameters depicted in Figure S7, we found that a small fraction (0.12) of the parameter range depicted in the figure failed to reject the null hypothesis at P < 0.01. The succinct conclusion from this simulation effort is that the magnitude of changes in allele frequencies seen in the ABS and PLE subpopulations is consistent with a simple demographic model with plausible magnitudes of change in population sizes and increased isolation between the two.

**Supplemental References**

Coulon, A., Fitzpatrick, J. W., Bowman, R., Stith, B. M., Makarewich, C. A., Stenzler, L. M., & Lovette, I. J. (2008). Congruent population structure inferred from dispersal behaviour and intensive genetic surveys of the threatened Florida scrub-jay ( *Aphelocoma cœrulescens* ). *Molecular Ecology*, *17*(7), 1685–1701. doi: 10.1111/j.1365-294X.2008.03705.x

Kapusta, A., Suh, A., & Feschotte, C. (2017). Dynamics of genome size evolution in birds and mammals. *Proceedings of the National Academy of Sciences*, *114*(8), E1460–E1469. doi: 10.1073/pnas.1616702114

Meyermans, R., Gorssen, W., Buys, N., & Janssens, S. (2020). How to study runs of homozygosity using PLINK? A guide for analyzing medium density SNP data in livestock and pet species. *BMC Genomics*, *21*(1), 1–14. doi: 10.1186/s12864-020-6463-x

Narasimhan, V., Danecek, P., Scally, A., Xue, Y., Tyler-Smith, C., & Durbin, R. (2016). BCFtools/RoH: A hidden Markov model approach for detecting autozygosity from next-generation sequencing data. *Bioinformatics (Oxford, England)*, *32*(11), 1749–1751. doi: 10.1093/bioinformatics/btw044

Purcell, S., Neale, B., Todd-Brown, K., Thomas, L., Ferreira, M. A. R., Bender, D., … Sham, P. C. (2007). PLINK: A Tool Set for Whole-Genome Association and Population-Based Linkage Analyses. *The American Journal of Human Genetics*, *81*(3), 559–575. doi: 10.1086/519795

Szpiech, Z. A., Blant, A., & Pemberton, T. J. (2017). GARLIC: Genomic Autozygosity Regions Likelihood-based Inference and Classification. *Bioinformatics*, *33*(13), 2059–2062. doi: 10.1093/bioinformatics/btx102
